# Supplementary material for: Introduction of electronic death notification in Norway—Impact on diabetes mortality registration
Source: PLoS One. 2024 Dec 2;19(12):e0311106. doi: 10.1371/journal.pone.0311106 (PMC11611212; doi:10.1371/journal.pone.0311106)
Supplement: S1 File — (PDF) [file pone.0311106.s001.pdf]

S1: Age-standardized mortality rates per 100 000 in Norway 2017 - 2022. Deaths with autopsy are excluded. CI; confidence interval, DM; diabetes mellitus, T1DM; diabetes type-1, T2DM; diabetes type-2

**Year**	**2017**	**2018**	**2019**	**2020**	**2021**	**2022**
**All causes of death**	822.0	811.7	791.3	776.3	778.5	852.2
95% CI	813.7-830.4	803.5-819.9	783.2-799.3	768.4-784.2	770.7-786.3	844.1-860.3
Men	966. 6	948.3	925.6	905.2	889.5	994.9
95% CI	952.3-981.0	934.3-962.3	911.9-939.2	891.9-918.5	876.5-902.4	981.4-1008.5
Women	719.8	711.5	694.0	681.8	695.3	744.8
95% CI	709.8-729.9	701.6-721.4	684.2-703.7	672.28-691.3	685.7-704.9	734.9-754.7
**DM, all**	12.6	11.3	11. 9	14.9	14.8	15.8
95% CI	11.5-13.6	10.3-12.3	10.9-12.9	13.8-16.0	13.7-15.9	14.7-16.9
Men	14.4	14.8	15.9	18.2	19.7	21.2
95% CI	12.7-16.1	13.1-16.5	14.2-17.7	16.3-20.0	17.8-21.7	19.3-23.2
Women	11.0	8.7	9.0	12.3	11.2	11.7
95% CI	9.8-12.3	7.6-9.8	7.9-10.1	11.0-13.6	10.0-12.4	10.5-13.0
**T1DM**	0.8	1.1	10	1.4	2.3	2.1
95% CI	0.6-1.1	0.8-1.4	0.7-1.3	1.1-1.7	1.9-2.7	1.7-2.5
Men	1.0	1.3	1.2	1.6	2.3	2.8
95% CI	0.6-1.4	0.8-1.9	0.8-1.7	1.1-2.1	1.6-2.9	2.1-3.5
Women	0.7	1.0	0.8	1.2	2.2	1.5
95% CI	0.3-1.0	0.6-1.4	0.4-1.1	0.8-1.6	1.6-2.7	1.0-1.9
**T2DM**	5.1	4.5	5.8	9.0	10.5	12.3
95% CI	4.5-5.8	3.9-5.1	5.1-6.4	8.1-9.8	9.6-11.4	11.3-13.3
Men	6.1	6.2	7.6	11.2	14.8	16.8
95% CI	5.0-7.2	5.1-7.4	6.3-8.8	9.7-12.7	13.1-16.5	15.0-18.5
Women	4.3	3.4	4.5	7.3	7.5	9.0
95% CI	3.6-5.1	2.7-4.0	3.7-5.3	6.3-8.3	6.5-8.5	7.9-10.1
**DM-other**	6.6	5.7	5.2	4.6	2.0	1.5
95% CI	5.9-7.4	5.0-6.4	4.5-5.8	4.0-5.2	1.6-2.4	1.1-1.8
Men	7.3	7.2	7.1	5.4	2.7	1.7
95% CI	6.1-8.5	6.1-8.4	5.9-8.3	4.4-6.4	2.0-3.4	1.1-2.2
Women	6.0	4.3	3.7	3.8	1.5	1.3
95% CI	5.1-7.0	3.6-5.1	3.0-4.5	3.0-4.5	1.1-1.9	0.9-1.7
