# Supplementary material for: Introduction of electronic death notification in Norway—Impact on diabetes mortality registration
Source: PLoS One. 2024 Dec 2;19(12):e0311106. doi: 10.1371/journal.pone.0311106 (PMC11611212; doi:10.1371/journal.pone.0311106)
Supplement: S2 File — (PDF) [file pone.0311106.s002.pdf]

S2: Death certificates with diabetes mellitus (DM) as underlying cause of death, specified type of DM, type of death certificate submission, sex and mean age. Deaths with autopsy are excluded. T1DM; diabetes type-1, T2DM; diabetes type-2, pDC; paper death certificate, eDC; electronic death certificate, N; number, Y; years

**Year**		**2017**	**2018**	**2019**	**2020**	**2021**	**2022**
**Total DM, N**		565	519	559	712	722	787
	pDC, N	565	519	537	435	127	29
	eDC, N	0	0	22	277	595	758
	Men, N	266	280	308	366	399	448
	Mean age, Y	78.2	78.3	78.7	79.0	80.1	78.9
	Women, N	299	239	251	346	323	339
	Mean age, Y	84.5	83.7	83.5	84.1	83.9	83.9
**T1DM, N**		38	52	48	67	112	106
	pDC, N	38	52	46	31	12	2
	eDC, N	0	0	2	36	100	104
	Men, N	21	26	28	35	51	66
	Mean age, Y	69.5	72.3	70.6	72.6	72.7	70.1
	Women, N	17	26	20	32	61	40
	Mean age, Y	81.1	71.9	77.1	80.3	78.3	78.1
**T2DM, N**		229	206	271	428	511	608
	pDC, N	229	206	251	203	54	11
	eDC, N	0	0	20	225	457	597
	Men, N	111	113	144	220	293	346
	Mean age, Y	79.5	80.4	80.2	80.4	81.5	80.5
	Women, N	118	93	127	208	218	262
	Mean age, Y	85,5	84,2	83,9	84,2	85,4	84,8
**DM-other, N**		298	261	240	217	99	73
	pDC, N	298	261	240	201	61	16
	eDC, N	0	0	0	16	38	57
	Men, N	134	141	136	111	55	36
	Mean age, Y	78.4	77.7	78.7	78.2	79.2	79.3
	Women, N	164	120	104	106	44	37
	Mean age, Y	84.1	85.9	84.3	85.1	84.5	83.3
