# Supplementary material for: Introduction of electronic death notification in Norway—Impact on diabetes mortality registration
Source: PLoS One. 2024 Dec 2;19(12):e0311106. doi: 10.1371/journal.pone.0311106 (PMC11611212; doi:10.1371/journal.pone.0311106)
Supplement: S3 File — (PDF) [file pone.0311106.s003.pdf]

S3: Deaths by diabetes mellitus, according to place of death, type of diabetes and type of submission. Deaths with autopsy are excluded. T1DM; diabetes type-1, T2DM; diabetes type-2, pDC; paper death certificate, eDC; electronic death certificate, N; number

**Year**		**2017**	**2018**	**2019**	**2020**	**2021**	**2022**
**Hospital**	T1DM, N	13	16	12	12	22	29
	T2DM, N	39	42	58	68	98	104
	DM-other, N	32	28	35	28	14	11
	Total, N	84	86	105	108	134	144
	Ratio of all DM deaths, %	14.9	16.6	18.8	15.2	18.6	18.3
	pDC, N	84	86	104	64	18	3
	eDC, N (%)	0 (0)	0 (0)	1 (0)	44 (40.7)	116 (86.6)	141 (97.9)
**Other health institutions**	T1DM, N	18	26	26	34	63	53
	T2DM, N	154	128	167	264	315	376
	DM-other	185	147	126	120	47	36
	Total, N	357	301	319	418	425	465
	Ratio of all DM deaths, %	63.2	58.0	57.1	58.7	58.9	59.1
	pDC, N	357	301	303	245	68	11
	eDC, N (%)	0 (0)	0 (0)	16 (5.0)	173 (41.4)	357 (84.0)	454 (97.6)
**Private homes, other or unknown**	T1DM, N	7	10	10	21	27	24
	T2DM, N	36	36	46	96	98	128
	DM-other, N	81	86	79	69	38	26
	Total, N	124	132	135	186	163	178
	Ratio of all DM deaths, %	22.0	25.4	24.2	26.1	22.6	22.6
	pDC, N	124	132	112	126	41	15
	eDC, N (%)	0 (0)	0 (0)	23 (17.0)	60 (32.3)	122 (74.8)	163 (91.6)
