# Supplementary material for: Introduction of electronic death notification in Norway—Impact on diabetes mortality registration
Source: PLoS One. 2024 Dec 2;19(12):e0311106. doi: 10.1371/journal.pone.0311106 (PMC11611212; doi:10.1371/journal.pone.0311106)
Supplement: S4 File — (PDF) [file pone.0311106.s004.pdf]

S4: Diabetes mellitus (DM) as underlying cause of death (UCOD) after automatic processing of diagnoses from death certificates (DCs), according to use of original or changed diagnoses. In UCODs with original diagnoses, the position of DM type is specified. Deaths with autopsy are excluded.

T1DM; diabetes type-1, T2DM; diabetes type-2, pDC; paper death certificate, eDC; electronic death certificate,N; number

**Year**		**2017**	**2018**	**2019**	**2020**	**2021**	**2022**
**Original DM diagnoses as UCOD, total, N**		200	163	171	299	427	522
Ia	T1DM	3	2	2	3	11	6
	T2DM	7	4	5	21	21	27
	DM-other	12	5	5	5	5	9
	Ia, all	22	11	12	29	37	42
Ib	T1DM	8	5	5	6	33	25
	T2DM	22	21	28	54	83	103
	DM-other	44	43	32	32	20	12
	Ib, all	74	69	65	92	136	140
Ic	T1DM	0	1	4	14	13	18
	T2DM	3	5	10	23	60	83
	DM-other	17	7	9	7	0	8
	Ic, all	20	13	23	44	73	109
Id	T1DM	0	0	0	0	1	6
	T2DM	0	0	1	14	30	37
	DM-other	0	0	0	0	0	0
	Id, all	0	0	1	14	31	43
Part II	T1DM	5	6	2	11	20	24
	T2DM	35	25	38	79	111	151
	DM-other	44	39	30	30	19	13
	Part II, all	84	70	70	120	150	188
**Changed DM diagnoses as UCOD, total, N**							
	T1DM	22	38	35	33	34	27
	T2DM	162	151	189	237	206	207
	DM-other	181	167	164	143	55	31
	Changed, all	365	356	388	413	295	265
**Total DCs, N**		565	519	559	712	722	787
