# Supplementary material for: Introduction of electronic death notification in Norway—Impact on diabetes mortality registration
Source: PLoS One. 2024 Dec 2;19(12):e0311106. doi: 10.1371/journal.pone.0311106 (PMC11611212; doi:10.1371/journal.pone.0311106)
Supplement: S5 File — (PDF) [file pone.0311106.s005.pdf]

S5: Diabetes mellitus (DM) as underlying cause of death (UCOD) from death certificates (DCs), according to type of death certificate and the original position of diabetes type diagnoses. Deaths with autopsy are excluded. T1DM; diabetes type-1, T2DM; diabetes type-2, pDC; paper death certificate, eDC; electronic death certificate, N; number

**Type of diabetes and placement on the death certificate**		**2017**	**2018**	**2019**	**2020**	**2021**	**2022**
DM, only part I		403	360	405	518	526	562
	pDC	403	360	393	314	90	20
	eDC	0	0	12	204	436	542
DM, only part II		150	150	151	190	191	213
	pDC	150	150	141	118	36	9
	eDC	0	0	10	72	155	204
DM, both part I and II		12	9	3	4	5	12
	pDC	12	9	3	3	1	0
	eDC	0	0	0	1	4	12
T1DM	Only part I						
	pDC	28	30	40	24	7	2
	eDC	0	0	2	27	81	74
	Only part II						
	pDC	7	18	5	7	4	0
	eDC	0	0	0	9	19	28
	Both part I and II						
	pDC	3	4	1	0	1	0
	eDC	0	0	0	0	0	2
T2DM	Only part I						
	pDC	155	141	175	139	35	7
	eDC	0	0	10	165	327	425
	Only part II						
	pDC	69	63	75	62	19	4
	eDC	0	0	10	60	126	163
	Both part I and II						
	pDC	5	2	1	2	0	0
	eDC	0	0	0	0	4	9
DM-other	Only part I						
	pDC	220	189	178	151	48	11
	eDC	0	0	0	12	28	43
	Only part II						
	pDC	74	69	61	49	13	5
	eDC	0	0	0	3	10	13
	Both part I and II						
	pDC	4	3	1	1	0	0
	eDC	0	0	0	1	0	1
