# Supplementary material for: Introduction of electronic death notification in Norway—Impact on diabetes mortality registration
Source: PLoS One. 2024 Dec 2;19(12):e0311106. doi: 10.1371/journal.pone.0311106 (PMC11611212; doi:10.1371/journal.pone.0311106)
Supplement: S6 File — (PDF) [file pone.0311106.s006.pdf]

**S6:** Diabetes ICD-10 codes used in cases with diabetes mellitus (DM) as underlying cause of death. Deaths with autopsy ae excluded.

| **DM codes** | | **2017** | **2018** | **2019** | **2020** | **2021** | **2022** |
|---|---|---|---|---|---|---|---|
| E10.0 | Type 1 diabetes mellitus with coma | 0 | 2 | 3 | 0 | 3 | 1 |
| E10.1 | With ketoacidosis | 4 | 3 | 1 | 4 | 0 | 2 |
| E10.2 | With renal complications | 2 | 9 | 6 | 9 | 10 | 9 |
| E10.3 | With ophthalmic complications | 0 | 0 | 0 | 1 | 0 | 0 |
| E10.4 | With neurological complications | 0 | 1 | 0 | 1 | 2 | 0 |
| E10.5 | With peripheral circulatory complications | 1 | 1 | 5 | 1 | 4 | 5 |
| E10.6 | With other specified complications | 9 | 9 | 13 | 16 | 14 | 5 |
| E10.7 | With multiple complications | 12 | 17 | 12 | 23 | 55 | 66 |
| E10.8 | With unspecified complications | 1 | 1 | 1 | 4 | 10 | 12 |
| E10.9 | Without complications | 9 | 9 | 7 | 8 | 14 | 6 |
| E11.0 | Type 2 diabetes mellitus with coma | 2 | 1 | 1 | 3 | 3 | 3 |
| E11.1 | With ketoacidosis | 1 | 1 | 0 | 4 | 4 | 1 |
| E11.2 | With renal complications | 42 | 45 | 47 | 62 | 65 | 50 |
| E11.3 | With ophthalmic complications | 0 | 0 | 1 | 5 | 5 | 5 |
| E11.4 | With neurological complications | 0 | 0 | 3 | 3 | 0 | 2 |
| E11.5 | With peripheral circulatory complications | 13 | 12 | 18 | 22 | 10 | 23 |
| E11.6 | With other specified complications | 74 | 55 | 73 | 77 | 58 | 64 |
| E11.7 | With multiple complications | 35 | 41 | 64 | 154 | 271 | 332 |
| E11.8 | With unspecified complications | 2 | 1 | 1 | 18 | 30 | 57 |
| E11.9 | Without complications | 60 | 50 | 63 | 80 | 65 | 71 |
| E12.4 | Malnutrition-related diabetes mellitus with neurological complications | 0 | 0 | 0 | 0 | 3 | 1 |
| E12.6 | With other specified complications | 0 | 0 | 0 | 0 | 0 | 1 |
| E12.7 | With multiple complications | 0 | 0 | 0 | 1 | 1 | 2 |
| E12.9 | Without complications | 0 | 0 | 0 | 0 | 0 | 4 |
| E13.2 | Other specified diabetes mellitus with renal complications | 0 | 0 | 0 | 0 | 0 | 1 |
| E13.6 | With other specified complications | 0 | 0 | 0 | 0 | 1 | 2 |
| E13.7 | With multiple complications | 0 | 0 | 0 | 0 | 0 | 2 |
| E14.0 | Unspecified diabetes mellitus with coma | 0 | 0 | 0 | 1 | 0 | 0 |
| E14.1 | With ketoacidosis | 1 | 0 | 0 | 1 | 0 | 3 |
| E14.2 | With renal complications | 44 | 39 | 32 | 37 | 10 | 10 |
| E14.3 | With ophthalmic complications | 0 | 0 | 1 | 0 | 0 | 0 |
| E14.4 | With neurological complications | 1 | 1 | 0 | 3 | 0 | 0 |
| E14.5 | With peripheral circulatory complications | 16 | 11 | 12 | 7 | 5 | 2 |
| E14.6 | With other specified complications | 88 | 77 | 81 | 73 | 26 | 4 |
| E14.7 | With multiple complications | 41 | 47 | 44 | 29 | 18 | 23 |
| E14.8 | With unspecified complications | 2 | 1 | 0 | 1 | 6 | 6 |
| E14.9 | Without complications | 105 | 85 | 70 | 64 | 29 | 12 |
| Total | | 565 | 519 | 559 | 712 | 722 | 787 |
