# Supplementary material for: Introduction of electronic death notification in Norway—Impact on diabetes mortality registration
Source: PLoS One. 2024 Dec 2;19(12):e0311106. doi: 10.1371/journal.pone.0311106 (PMC11611212; doi:10.1371/journal.pone.0311106)
Supplement: Attachment — Submitted filename: Response to the Reviewers.docx [file pone.0311106.s007.docx]

PLOS ONE

Regarding: PONE-D-24-05293

Introduction of Electronic Death Notification in Norway - Impact on Diabetes Mortality Registration

Response to reviewers:

**Reviewer #1:** This study has been conducted in Norway to study the impact of introducing the e death certification system in 2018 and making the e notifications mandatory from 2022. The authors have compared the diagnosis of Diabetes as the underlying cause of death (UCOD) from 2017 to 2022. The study shows in increase of reporting diabetes as UCOD after the introduction of e notification in 2018 and becoming e DC mandatory in 2022.
The rationale for the study is clear and valid. The authors have used a technically suitable protocol and a feasible methodology to achieve the study's aims effectively. I congratulate the authors for doing this all-important study after a change in the death certification system.
However, I have the following comments about their methods and the study.

1. In a paper-based certification system, the doctors will enter cause of death data in free text in parts 1 and 2 of the WHO recommended format in handwritten format. In an e death certification system doctors will enter free text in the same WHO recommended format but the causes are type written. The main advantages are clarity due to lack of illegible handwriting, adherence to the prescribed format, and following the guidelines (e.g. One condition per line, sequence, mandatory time intervals, etc.) In this study, the authors do not discuss the immediate advantages of e-certification.

*Information about the main and immediate advantages of an electronic death certification system are now incorporated in the Introduction, see lines 65-69 in the manuscript.*

2. The main benefits the authors discuss are not due to e-certification but to introducing a drop-down menu of ICD-10 categories and codes. Drop-down menus are known to cause systematic errors in certification and coding. Using a drop-down menu, the certifiers bypass volume 3 of ICD-10 and directly assign ICD codes from the tabular list, bypassing exclusion criteria and other coding conventions in volume 1. This is an inherent error in using drop-down menus.

*The drop-down menu in the Norwegian online system allows searches both by text and codes, as text (with* codes*) appears while writing, thus preventing bypassing of volume 3 of the ICD-10, a possible bias discussed by reviewer #1. We have clarified the advantages and the structure of the drop-down menu in the Introduction and expanded the discussion on any negative effects in the Discussion. See lines 66-69 and 285-299 iin the manuscript.*

3. The authors have not described how the coding was done before the certification system. It may be Iris's text entry mode based on a dictionary. With the introduction of e certification, now they use Iris in the code entry mode. But the primary code assignments to individual causes of death from the drop-down menu may not be the best code choices for assignment.

*In Norway, IRIS/ACME has been a fully automated coding system since 2011 and the registration system has remained basically the same, before and after introduction of the online system In the manual system, the full text, as written by the physician, was translated into codes by IRIS. As described in 2), Iris is used in the text entry mode, as a dictionary. When text is selected from the drop-down menu in the electronic reporting system, the chosen diagnoses/conditions appear and are linked to ICD-codes. Thus, the only difference between the manual and the online system is that the manual step of entering written text is bypassed. We hope the revised text in the Introduction and Discussion is clarifying enough. See lines 91-102 and 285-299 in the manuscript.*

4. Reduction of ill-defined ICD codes is a positive finding. However, the increase in specific diabetes mellitus codes does not necessarily indicate improved certification and coding practices because the codes are necessarily from the drop-down menu.

*As mentioned above, the drop-down menu allows for dictionary searches. We have introduced a discussion on the possibility of position bias, for which we have no indication, in the Discussion. See lines 285-299 in the manuscript.*

5. If the study's objective is to investigate the certification practices after e certification system, the authors must conduct a medical record review to validate e death certificate findings with the hospital medical record content. If such a validation proves an increased sensitivity and Positive Predictive Value, it will be a proof of improved cause of death data following e certification.

*We fully agree that this would be of great value. However, it would have required a different set-up than in the present study. We already know that death certification is hampered by a certain level of uncertainty, mostly due to lack of training by the physicians or incorrect clinical information*, and instruments have been developed to surpass the most common failures (amongst them Iris/ACME). The purpose of the present study however, was not to quality control the diagnoses listed on the death certificates, but to look for changes in the statistics after introduction of online submission.*

**See: Eng HM, L Ellingsen C, Pedersen AG, Alfsen GC. Cause of death certificates in nursing homes: Does quality matter? A retrospective review from two counties in Norway. Scand J Public Health. 2023 Jul 26:14034948231187512. doi: 10.1177/14034948231187512. Epub ahead of print. PMID: 37491994.*

6. I would suggest the authors to screen death certificates for the reduction of common errors in medical certification. The most common errors are,
a. Reporting multiple causes per line
b. Absence of time intervals
c. Illegibility
d. Use of non-standard abbreviations
e. Reporting ill-defined causes and modes of dying as underlying cause of death
f. Incorrect causal sequence in part 1 of the death certificates.

*A study based on the string of codes after automated analyses, as we have performed, has limitations. Thus, we are not able to analyse for errors regarding the points a- d.
e) The reporting of ill-defined causes is already part of our study. As we have analysed for deaths by diabetes only, our data do not contain information on deaths registered as by ill-defined causes. However, a recent publication on garbage codes in Norway has interesting information on this*.
f) We have looked at the position of diabetes diagnoses, but only in regard to position I or II, and after automated analyses. A study of incorrect or correct causal sequences would require access to the original death certificates, which we didn’t have.*

*** see *Ellingsen CL, Alfsen GC, Ebbing M, Pedersen AG, Sulo G, Vollset SE, Braut GS. Garbage codes in the Norwegian Cause of Death Registry 1996-2019. BMC Public Health. 2022 Jul 7;22(1):1301. doi: 10.1186/s12889-022-13693-w. PMID: 35794568; PMCID: PMC9261062.*

7. The authors can still study the impact on diabetes mortality registration. However, while doing that you may identify the drop-down menu in e certificate as a limiting factor.

*We agree and have discussed this limitation. See lines 285-299 in the manuscript.*

**Reviewer #2:** This article addresses an important topic - that of the impact of the introduction of electronic death notification on reporting of diabetes mortality. However there are several serious deficiencies in how the study is presented that need to be addressed if it is to be suitable for publication.

The main issue is that many of the results that are described in the Abstract and the main text - specifically the relative risks from the generalized linear models - are not presented in any tables.

No Table is referenced in the main text and the results are not presented in any of the tables that are shown. This means that it is not possible to assess the results adequately. Presentation of such results in a table is a pre-requisite for such a manuscript.

*We have rewritten parts of the manuscript accordingly. Quality variables have been systematically described in the Materials and Methods and the results collected in a new Table 2, with relevant statistics. See lines 128-141 and 211-230 in the manuscript.*

The manuscript does not adequately provide a rationale for the study and the knowledge gap that the study is filling. Why is accurate reporting of diabetes important? For example, what is its ranking as a leading cause of death or disease burden in Norway, what is its impact on the health system? There also needs to be more detailed exploration of the literature on this topic and description of the existing knowledge gap, so that the reader understands the contribution that the manuscript makes.

*The mentioned topics are now included in the Introduction. See lines 103-111 in the manuscript.*

Is there a variable about region of the country that could be included in the models?

*We did not look at regional differences in the present study.*

There also needs to be more background about the introduction of the electronic death certificate - how it was rolled-out, how doctors were trained, etc - as well as the process of a doctor completing the electronic death certificate and submitting the certificate.

*The Introduction has been expanded accordingly. See lines 74-79 and 80-90 in the manuscript.*

What does it mean by "variable for year of death was centered"? This should be clearer.

*This is reformulated in the section on statistical method. See lines 150-152 in the manuscript.*

The information in Lines 159-163 is not really important and can be deleted or significantly reduced.

*We agree. Detailed information on mortality rates regarding cause of death, gender and age has been removed from the text, but can easily be found in the supplementary material. For the same reason, we also have deleted the next sentence, on place of death.*

The writing needs to be improved. There are several places with terminology that needs to be more detailed (Line 31 "International classification of diseases 10 codes" should be "10th Revision") or there are errors (Line 36 eCD instead of eDC), I44.9 for cardiac arrest should be I46). Please review carefully.

*The manuscript has been reviewed carefully and all errors should be corrected. In regard to I46.9: We are grateful for the thorough reading! It proved out that this was not only a typing error but repeated itself also in our calculations, which is why the results in the new Table 2 now differs slightly from the original manuscript.*

What is "Id" in Line 240?

*Id is related to extension of the chain of events. The heading is changed accordingly, to make it more readable. See line 276 in the manuscript.*

Line 232: "January 1st2022" should be corrected.

*This has been corrected.*

**Reviewer #3:** While commend the efforts of the authors, the following comments are given with the hope that these will be beneficial for them.

Major comments:
1. The introduction section needs to be enriched with more details; more information of the paper-based system used, why countries are shifting to electronic systems, brief account on what is meant by quality of mortality registration and data on diabetes burden (e.g. prevalence) in the Norway.

*The Introduction is expanded accordingly, with more information about the death certification systems, mortality registration and disease burden. See lines 74-111 in the manuscript.*

2. It would be better to clearly mention and operationalize the variables assessed for quantity and quality in one place in the methodology.

*We have systematised all quality variables in the Materials and Methods and collected the results in a new Table 2. See lines 128-141 (and 211-230) in the manuscript.*

3. More details are needed in the methods section on how the data analyses were done. As an example how were verification done (e.g. whether two investigators analyzed the data independently etc.)

*We have worked with absolute data, and verification procedures as described above, are thus not relevant.*

4. It would be better if a table can be inserted to show the relative risk statistics and p values mentioned in abstract and in results.

*We agree. See new Table 2. See line217 in the manuscript.*

Minor comment
-There is a confusion on the uniformity of the abbreviations used. Examples include:
1. What is meant by "CDs" in the last sentence of the results in abstract
2. eCD and eDC in methods of abstract

*Spelling errors have been corrected.*
