# Supplementary material for: Introduction of electronic death notification in Norway—Impact on diabetes mortality registration
Source: PLoS One. 2024 Dec 2;19(12):e0311106. doi: 10.1371/journal.pone.0311106 (PMC11611212; doi:10.1371/journal.pone.0311106)
Supplement: Attachment — Submitted filename: Response to Reviewers.docx [file pone.0311106.s008.docx]

To the editors.

Regarding PONE-D-24-05293R1

We thank you for thorough reviews with valuable comments, undoubtedly contributing to raise the quality and ease the readability of our manuscript.

Changes in the present (and hopefully final) revision are listed below:

1. The reference list has been reviewed in detail and should now be in par with the guidelines of the journal.
2. A "total" row to the table in S6 file (i.e. in supporting information) is added.


**Comments from reviewers:**

Reviewer #1: Thanks for responding to my review questions.
My main concerns were related to the use of a drop-down menu to record diagnoses and assign ICD codes in the eDC and the use of Iris in the code-entry mode for selecting the underlying cause. My comments were based on the following section of the methodology (lines 80 – 88)
‘The processing of DCs by the NCoDR remains unchanged. DCs completed from the drop-down menu are automatically assigned with ICD-codes and in most causes do not require further handling. Causes of death in pDCs og eDCs with free text are manually registered. The resulting string of codes reflects the order of diseases or conditions given in the DC, regardless of type of submission and/or use of free text or drop-down menu. The string of codes from each DC is subject to automatic processing by an automated coding system (Iris) based on the Automatic Classification of Medical Entry software (ACME), which selects UCOD according to rules and guidelines provided by WHO (ICD-10) (10, 11, 12). Complex cases are reviewed by specially trained staff from the NCoDR before selection of UCOD in Iris.’
As I explained before, the use of drop-down menus and the selection of ICD codes from the drop-down can lead to systematic errors in certification and coding.
However, the resubmission explains the certification and coding process (Lines 91-100), and the clarity has been improved.
‘ After submission, the processing of DCs by the NCoDR remains unchanged. eDCs completed from the drop-down menu are automatically assigned with ICD-10 codes. Allocations of ICD-codes in death certificates with free text are achieved after manual registration of diagnoses, by means of handling by the automated coding system (Iris) based on the Automatic Classification of Medical Entry software (ACME) (13, 14). The resulting “string of codes” reflects the order of diseases or conditions given in the DC, regardless of type of submission and/or use of free text or drop-down menu. The string of codes from each DC is subject to renewed automated processing by Iris/ACME, which selects UCOD according to rules and guidelines provided by WHO (ICD-10) ( 15). Automated processing may lead to changes or specifications of the original ICD-10 code on the DC, dependent on the setting of diagnoses on the DC.’

We are pleased to see that this section now is satisfactory.

In your response to reviewer question 6, you mentioned, ‘A study of incorrect or correct causal sequences would require access to the original death certificates, which we didn’t have.’ I would appreciate it if you could explain the difference between eDC and the original death certificate. How could the original death certificate differ from eDC? For what purposes do you use the original death certificate?

As explained in the introduction (lines 88-89), the physicians have the possibility of free text also in the electronic version of DCs. Certificates with free text require manual handling. Thus, the “string of codes” after handling by the NCoDR may not be fully identical to the submitted, original version, even in eDCs. Unfortunately, data on how often free text is used in electronic submitted DCs is presently not recorded.

We fully agree that studies on the occurrence of incorrect causal sequence in part 1 of the death certificates would be an excellent quality measure. A complicating factor is that the IT-system used in NCoDR does not allow for the detection of which WHO-rules are used when selecting UCOD. We believe that analyses as suggested in question 6-f also will require a larger material than diabetes deaths only. We are presently planning to look into this, using data from all deaths. We hope to present these data by next year.

Reviewer #3: The author has sufficiently and clearly addressed the comments provided in the previous review. The introduction is now more coherent, effectively emphasizing the significance of transitioning to the electronic death certificate system in Norway. The quality of the manuscript has been greatly improved and I extend my congratulations to the authors.

We are grateful for constructive comments to our manuscript.

Reviewer #4: •This study conducted in Norway examines the impact of introducing the electronic death certification system in 2018 and making e-notifications mandatory in 2022. The authors compared the diagnosis of diabetes as the underlying cause of death (UCOD) from 2017 to 2022, finding an increase in reporting diabetes as UCOD after these changes. The study's rationale is clear and valid, highlighting the importance of improving diabetes management in Norway. The authors employed a suitable protocol and feasible methodology to effectively achieve the study's aims. I commend the authors for this important study following changes in the death certification system. However, I have the following comments:

•Since 5% of submissions in 2022 were paper-based, it is important to mention how many of these were diabetes-related. If the percentages of paper-based diabetes-related deaths in 2017 and 2022 are similar, it would suggest that the sample is representative across both years and submission methods. The authors should also clearly clarify whether the 5% paper-based diabetes-related deaths in 2022 were excluded from the analysis((127).

The numbers of death certificates on paper for all deaths are given in table 1 (2022: n=1995 / 4,7% of all deaths). For diabetes, relations of diabetes deaths and type of death certificate submission are illustrated in Figure 1, and the data given in supporting information S2 (pDC 2022: n=29 / 3,7% of diabetes deaths). As discussed in lines 200-205, this difference was significant.

Due to the small number of pDCs in 2022, we did not exclude diabetes deaths reported on paper in 2022 (2022: 29/ 1995=1.45 %) We have added a sentence in Statistical methods explaining this (see line 154).

•Minor correction in line 59: “freetext” should be corrected to "free text" if it is not one word.

Corrected.

•In line 100, define what T1DM stands for (Type 1 Diabetes Mellitus).

As this is an introductory example, abbreviations have been removed.

There are several errors in Table 2 (line 217). Although it mentions -N (%), only numbers are provided without percentages.

We apologize for having forgotten to add percentages. These are now added.

The position of UCOD in part I, RR: 1.03 CI: 1.00-1.05, should be checked for significance, as a CI including 1 is not significant.

We are grateful for this important correction and have changed the manuscript accordingly (lines 48-49 (abstract), table 2, line 221 and 225 (results), and line 253 (Key results)).

Additionally, the mean number of diagnoses per DC and the corresponding RR is not mentioned. Results of Table 2 are not adequately interpreted.

Under statistical methods we explain the different methods used for the different quality variables. For variable 4, where data were normally distributed, we used a two-sample t-test, and linear regressions to estimate additive changes and CI. To clarify this, we have revised the language under statistical methods to “a two-independent sample t-test” instead of “a two-sample t-test”. See line 158.

•There was no targeted training of physicians in connection with the introduction of the new submission system (line 76). It can be included as a recommendation to provide such training to further improve the system.

The online cause of death submission system introduced in Norway was thought to be intuitive, requiring no further training. However, we fully agree that training systems for improving cause of death registrations by physicians should be introduced. We have suggested to the Norwegian health authorities to make the existing online course mandatory. We have added a recommendation on this in the discussion (line 275-278).

Attached:

Manuscript with and without changes (“Revised Manuscript with Track Changes” and “Manuscript”)

Figure 1 (no changes)

Supporting information (S1 File-S5 File (no changes) and S6 File added with a "total" row)

We hope that the answers to the reviewers’ questions and that the revision of the script as well are satisfactory.

Best regards,

Hanna Maria Eng
