# Supplementary material for: Introduction of electronic death notification in Norway—Impact on diabetes mortality registration
Source: PLoS One. 2024 Dec 2;19(12):e0311106. doi: 10.1371/journal.pone.0311106 (PMC11611212; doi:10.1371/journal.pone.0311106)
Supplement: Attachment — Submitted filename: comments.docx [file pone.0311106.s009.docx]

**Thank you for addressing the previous comments. Please find the revised comments below:**

1. **Line 143:**
   - **Comment:** Please ensure that abbreviations are not used at the beginning of sentences. The revised text should fully spell out terms to enhance clarity and readability.
2. **Table 2, Line 218:**
   - **Comment:** The confidence interval (CI) for Underlying Cause of Death (UCOD) listed as 1-1.05 is not statistically significant. Please review and correct this information to accurately reflect statistical significance.
