# Supplementary material for: Introduction of electronic death notification in Norway—Impact on diabetes mortality registration
Source: PLoS One. 2024 Dec 2;19(12):e0311106. doi: 10.1371/journal.pone.0311106 (PMC11611212; doi:10.1371/journal.pone.0311106)
Supplement: Attachment — Submitted filename: Response to Reviewers.docx [file pone.0311106.s010.docx]

To the editors.

Regarding PONE-D-24-05293R1

We thank you for thorough reviews with valuable comments, undoubtedly contributing to raise the quality and ease the readability of our manuscript.

**Comments from reviewers:**

**Reviewer #1:** Thanks for submitting your responses to the reviewer questions.
While accepting you submission, I would like to draw your attention to some of your responses. In your response, you have said that all death certificates completed using free-text require manual handling. That is not correct. I'm sure you use a Iris dictionary for the purpose of batch processing in the text-entry mode. Iris is able to code death certificates containing free text in batch processing. We are grateful for these comments. To clarify this, we have chosen to rephrase this part in the manuscript (line 91-102 in Manuscript with Track Changes).

You have also mentioned that your IT system doesn't indicate which rules were used during mortality coding. However, Iris MUSE (Multi Causal and Unicausal Selection Engine) provides the users a full explanation of coding rules applied and the entire coding process. We are sorry that our dataset did not allow for this type of statistics.

**Reviewer #4:** Thank you for addressing the previous comments. Please find the revised comments below:
1. Line 143:
o Comment: Please ensure that abbreviations are not used at the beginning of sentences. The revised text should fully spell out terms to enhance clarity and readability.

Corrected (line 147 in Manuscript with Track Changes).

2. Table 2, Line 218:
o Comment: The confidence interval (CI) for Underlying Cause of Death (UCOD) listed as 1-1.05 is not statistically significant. Please review and correct this information to accurately reflect statistical significance.

For the record, statistical analyses were run again.

In this regard, we discovered a typing error from the previous run. We apologize for this and are grateful for this correction.

The result is unchanged in terms of statistical significance (p>0.05).

The correct results are RR=1.10, CI: 0.96-1.26, p=0.152.

We have corrected the values in table 2.

We hope that the answers to the reviewers’ questions and the revision of the script are satisfactory.

Attached:

Manuscript with and without changes (“Revised Manuscript with Track Changes” and “Manuscript”)

Figure 1 and supporting information (S1 File-S6 File) (no changes)

Best regards,

Hanna Maria Eng
